# Liver Cholesterol Overload Aggravates Obstructive Cholestasis by Inducing Oxidative Stress and Premature Death in Mice

**DOI:** 10.1155/2016/9895176

**Published:** 2016-08-21

**Authors:** Natalia Nuño-Lámbarri, Mayra Domínguez-Pérez, Anna Baulies-Domenech, Maria J. Monte, Jose J. G. Marin, Patricia Rosales-Cruz, Verónica Souza, Roxana U. Miranda, Leticia Bucio, Eduardo E. Montalvo-Jave, María Concepción Gutiérrez-Ruiz, Carmen García-Ruiz, José C. Fernández-Checa, Luis Enrique Gomez-Quiroz

**Affiliations:** ^1^Postgraduate Program in Experimental Biology, DCBS, Universidad Autónoma Metropolitana Iztapalapa, 09340 Mexico City, DF, Mexico; ^2^Health Science Department, Universidad Autónoma Metropolitana Iztapalapa, 09340 Mexico City, DF, Mexico; ^3^Translational Research Unit, Medica Sur Clinic and Foundation, 14050 Mexico City, DF, Mexico; ^4^Department of Cell Death and Proliferation, Instituto Investigaciones Biomédicas de Barcelona, CSIC, Barcelona and Liver Unit-Hospital Clinic-IDIBAPS and Centro de Investigación Biomédica en Red (CIBERehd), 08036 Barcelona, Spain; ^5^Laboratory of Experimental Hepatology and Drug Targeting (HEVEFARM), University of Salamanca, Centro de Investigación Biomédica en Red (CIBERehd), Instituto de Investigación Biomédica de Salamanca (IBSAL), 37007 Salamanca, Spain; ^6^Red “Fisiopatología de Enfermedades Hepáticas” PRODEP-SEP, 09340 Mexico City, DF, Mexico; ^7^General Surgery Service 304, General Hospital of Mexico, UNAM School of Medicine, 06726 Mexico City, DF, Mexico; ^8^Research Center for Alcohol Liver and Pancreatic Diseases and Cirrhosis, Keck School of Medicine, University of Southern California, Los Angeles, CA, USA

## Abstract

Nonalcoholic steatohepatitis is one of the leading causes of liver disease. Dietary factors determine the clinical presentation of steatohepatitis and can influence the progression of related diseases. Cholesterol has emerged as a critical player in the disease and hence consumption of cholesterol-enriched diets can lead to a progressive form of the disease. The aim was to investigate the impact of liver cholesterol overload on the progression of the obstructive cholestasis in mice subjected to bile duct ligation surgery. Mice were fed with a high cholesterol diet for two days and then were subjected to surgery procedure; histological, biochemical, and molecular analyses were conducted to address the effect of cholesterol in liver damage. Mice under the diet were more susceptible to damage. Results show that cholesterol fed mice exhibited increased apoptosis and oxidative stress as well as reduction in cell proliferation. Mortality following surgery was higher in HC fed mice. Liver cholesterol impairs the repair of liver during obstructive cholestasis and aggravates the disease with early fatal consequences; these effects were strongly associated with oxidative stress.

## 1. Introduction

Obesity is an epidemic problem; it has become a global health concern affecting rich and poor countries [1]. Obesity is the primary cause of nonalcoholic fatty liver disease (NAFLD), the commonest disease that affects liver function. It is highly prevalent and results from excessive fat accumulation, particularly free fatty acids, triglycerides (TG), and cholesterol [2], inducing a wide range of biochemical and clinical consequences leading to sensitization to damage and running to progressive disease stages such as nonalcoholic steatohepatitis (NASH), and fibrosis [3]. While the two-hit hypothesis posits that fat accumulation is key for progressive NAFLD, some of our studies have suggested that the kind of lipid rather than the amount of fat determines the susceptibility to secondary hits, including inflammatory cytokines such as the TNF-*α* family [4].

Although cholesterol is a critical component of membrane bilayers, its accumulation disrupts membrane fluidity or dynamics and promotes cellular dysfunction that could lead to disease progression [5]. The liver plays key role in the maintenance of cholesterol homeostasis, whose levels are determined by* de novo* synthesis and supply from the diet by serum lipoproteins [6]. Increased cholesterol deposition in the liver, in particular its accumulation in mitochondria, has emerged as one of the main toxic lipids in NAFLD due to cholesterol dependent mitochondrial dysfunction and sensitization to oxidative stress and inflammatory cytokines secondary to mitochondrial GSH depletion [4, 7–9]. The sensitization to the damage mediated by cholesterol overload can occur independently of nutritional oversupply as ob/ob mice and Niemann-Pick type C1- (NPC1-) lacking mice exhibit increased mitochondrial cholesterol accumulation and sensitization to inflammatory cytokines-mediated oxidative stress and cell death [4]. In line with these findings, there has been evidence that cholesterol intake increases the risk and severity of NAFLD and that patients with NASH have a higher expression of StARD1, a cholesterol transporting protein that regulates mitochondrial cholesterol homeostasis [8, 10, 11].

Despite this knowledge, the role of hepatic cholesterol accumulation in cholestatic liver disease has not been thoroughly examined. Hepatic accumulation of bile acids due to impairment in bile flow is central to the pathogenesis of cholestasis liver disease and leads to hepatic injury and, in severe cases, organ failure [12]. Bile acids' accumulation exerts noxious cellular events, ranging from oxidative stress and inflammation to apoptosis and necrosis [13, 14], leading to acute liver toxicity, proliferation of bile ducts, and fibrosis that eventually progresses to cirrhosis and liver failure. Moreover, it has been reported that patients with NAFLD exhibit alterations in bile acids homeostasis [15], suggesting that hepatic steatosis may determine the clinical presentation of cholestatic liver disease.

In the present work we addressed the impact of liver cholesterol overload in the BDL model of obstructive cholestasis. Our data show that nutritional hepatic cholesterol accumulation sensitizes to BDL-mediated liver injury and death.

## 2. Material and Methods

### 2.1. Animal Models

C57BL/6 male mice (8–10 weeks old) were purchased from Jackson Laboratory (Bar Harbor, Maine, USA) and were maintained in pathogen-free conditions with controlled temperature and humidity on a 12 h light-dark cycle in the animal care facility at the Universidad Autónoma Metropolitana and the Medical School of Universitat de Barcelona. The experimental protocols used were approved and performed in accordance with the Animal Care Committee of the University of Barcelona.

### 2.2. Experimental Design

Forty C57BL/6 mice were separated into two diet groups; the first one was fed with a high cholesterol (HC) (2% cholesterol, 0.5% sodium cholate) diet for two days as previously reported [4, 16]; the second group was fed with control standard Chow diet for the same period of time. Animals received water* ad libitum*. Afterwards, each group was subject to bile duct ligation (BDL) surgery or control Sham procedure. Five mice per time were sacrificed at days 1, 2, and 3, after surgery as depicted in Supplementary Figure  1(A) of the Supplementary Material available online at http://dx.doi.org/10.1155/2016/9895176. Five Sham animals were sacrificed only at day three. Liver tissue and serum were recovered for analysis. Six extra animals were used to figure out the effect of sodium cholate alone; we used a diet supplemented with 0.5% of the salt for 2 and 30 days (three mice per time) and liver gross inspection and AST and ALT serum activities were also determined (Supplementary Figures  1(B) and 1(C)).

### 2.3. Bile Duct Ligation Surgery

Surgery was performed under aseptic conditions. Anesthesia was induced with 2% isoflurane (Abbott Laboratories #B506) and 2-liter/min oxygen flow for a mouse of 25 g body weight. After median laparotomy, the common bile duct was ligated close to the liver* hilus*, below the bifurcation with 4-0 silk suture (Ethicon), to impede bile flow. The gallbladder was left intact. The abdominal muscle was sutured with a 5-0 surgical silk suture and the wound was closed with surgical staples. Control mice underwent a Sham surgery without BDL. All animals were allowed to wake up on a heating pad, and analgesia was given to all animals.

### 2.4. Determination of Liver Function Tests

Blood samples were obtained from the portal vein under isoflurane anesthesia before sacrifice. Serum levels of alanine aminotransferase (ALT), aspartate aminotransferase (AST), and alkaline phosphatase (ALP) were determined by the automated method using Reflovet Plus (Roche, Mannheim, Germany).

### 2.5. Histology and Immunohistochemical Studies

Liver tissue was fixed in 10% formalin/phosphate-buffered saline, dehydrated in alcohols, incubated in xylene, and embedded in paraffin. Paraffin molds containing liver sections were cut into 5 *μ*m sections and mounted on HistoGrip-coated slides. H&E was performed following standard procedures. Apoptosis was addressed by TUNEL detection kit (Trevigen Inc.) according to the manufacturer's protocol. Oil Red O (ORO) solution was used to detect neutral lipids in tissue.

For the immunohistochemistry of Ki67, paraffin sections were antigen unmasked with citrate buffer. Endogenous peroxidases were blocked with 3% H_2_O_2_; afterward slides were incubated with primary antibody overnight in a wet chamber at 4°C. After rinsing with PBS 1x, the slides were incubated with a biotinylated antibody for 45 min in a wet chamber and developed with the ABC kit with peroxidase substrate (DAB) and peroxidase buffer. After rinsing with tap water, slides were counterstained with hematoxylin. For filipin staining, frozen sections were fixed for 1 h at room temperature with formalin 10%, washed with PBS, and incubated with 0.2 mg/mL filipin over night at 4°C protected from light. After 3 final washes in PBS, tissues were mounted and confocal images (Carl Zeiss, LSM 780 multiphoton, Jena, Germany) were collected using UV excitation.

### 2.6. Cholesterol Determination

Ten mg of liver tissue or 200 *μ*L of serum was saponified with alcoholic KOH in a 60°C heating block for 15 min. After the mixture had cooled, 2 mL of hexane and 600 *μ*L of distilled water were added and shaken to ensure complete mixing. Appropriate aliquots of the hexane layer were evaporated under nitrogen and used for cholesterol measurement with* O*-phthalaldehyde reagent (Sigma-Aldrich) dissolved in acetic acid; after that sulfuric acid was added and then read at 550 nm in the spectrophotometer.

### 2.7. Triglycerides Determination

Triglyceride (TG) content was determined using the triglycerides determination kit (Sigma-Aldrich, San Louis, MO) following the manufacture's instructions.

### 2.8. Western Blot

Western blotting was performed following the protocol previously reported by Clavijo-Cornejo et al. [17]. Briefly, total proteins were isolated from liver homogenate with T-PER (Pierce) extraction reagents, supplemented with protease and phosphatase cocktail inhibitors (Roche Inc.). One hundred *μ*g of total protein was separated on precast 4–20% gels (Invitrogen), transferred to polyvinylidene difluoride membranes (PVDF, Invitrogen), and probed with different antibodies as shown in Supplementary Figure 2. Membranes were incubated with anti-mouse or anti-rabbit horseradish peroxidase-conjugated secondary antibody depending on the origin of the primary antibody. Immunoreactive bands were identified with ECL-Plus Western blotting detection reagents (GE Healthcare). Equal loading was demonstrated by probing the same membranes with actin antibody (Sigma-Aldrich).

### 2.9. Caspase 3 Activity

Tissue was lysed in reaction buffer and incubated for 30 min on ice. For each reaction, caspase 3 synthetic fluorogenic tetrapeptide substrate (Ac-DEVD-AMC) was added (BD Pharmingen) and then the tissue lysate; after that samples were incubated for one hour. The amount of AMC released was measured by fluorometry at an excitation wavelength of 380 nm and an emission wavelength of 420 nm.

### 2.10. In Situ ROS Determination

Animals were sacrificed in parallel exclusively for* in situ* ROS determination. Fresh tissue was rapidly sectioned, frozen in liquid nitrogen, and embedded in optimum cutting temperature compound (OCT, Sakura Finetek, Torrance, CA); subsequently, 8 *μ*m frozen sections were obtained in a cryostat (Leica CM-3050S, Heerbrugg, Switzerland) at −20°C and the slides were immediately incubated for 15 min, in the dark, at room temperature with either DCFH (5 *μ*M), a cell-permeable nonfluorescent probe that is deesterified intracellularly and converted to the highly fluorescent 2′,7′-dichlorofluorescein upon oxidation by ROS, particularly peroxides (H_2_O_2_), or with dihydroethidium* *(DHE, 50 *μ*M) for determination of superoxide anion radical (O_2_^−^) detecting ethidium fluorescence. Samples were covered and observed using a confocal microscope at excitation and emission wavelengths of 480 and 520 nm, respectively, for DCFH and 485 and 570 nm, respectively, for DHE-derived ethidium fluorescence, as previously we reported [18].

### 2.11. Glutathione (GSH) Measurements

GSH levels in tissue homogenates were analyzed by HPLC as previously we reported [18].

### 2.12. Bilirubins Determination

Bilirubins content was assayed by using Bilirubin Jendrassik-Grof FS kit (DiaSys Inc.) following the manufacturer's instructions.

### 2.13. Statistical Analysis

Data are presented as mean ± SEM for at least four different animals, and each experiment was carried out in triplicate. Comparisons between groups were performed using the ANOVA test followed by a* post hoc* Bonferroni test.

Additional materials and methods can be found in Supplementary Material.

## 3. Results

### 3.1. A High Cholesterol Diet Induces Liver Steatosis

**Figure 1:**
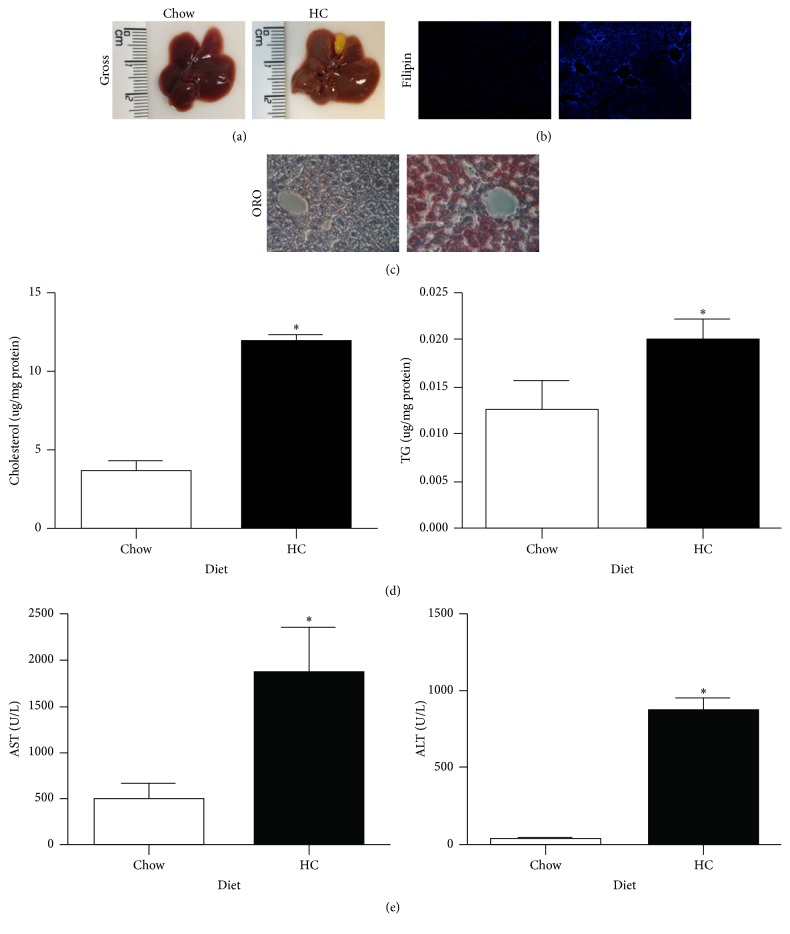
A high cholesterol diet induces liver steatosis. (a) Macroscopic liver inspection of standard control Chow diet (Chow) and high cholesterol (HC) diet. (b) Free cholesterol determination by filipin. (c) Neutral lipid determined by Oil Red O staining (ORO) in Chow and HC liver sections. (d) Biochemistry determination in liver tissue of total cholesterol and triglycerides (TG) content. (e) Liver function tests: aspartate aminotransferase (AST) and alanine aminotransferase (ALT). Each column represents mean ± SEM of three independent experiments. Differences were considered significant at ^*∗*^ *p* ≤ 0.01 versus Chow. Images are representative of at least three independent experiments. Original magnification: 200x.

To address the role of cholesterol hepatic overloading in BDL-mediated liver injury, we fed mice with a diet enriched in cholesterol (HC), as previously described [4, 16]. As seen, HC fed mice exhibited the characteristic pale color of the steatotic liver compared to mice fed with the control Chow diet (Figure 1(a)). In addition, filipin and ORO staining in liver tissue revealed a high content of both free cholesterol and neutral lipids (Figure 1(b)), findings that were biochemically confirmed in tissue homogenates (Figure 1(c)). Moreover, in line with findings in intact liver, isolated hepatocytes from HC fed mice revealed increased filipin and ORO staining (Supplementary Figure 3), confirming the accumulation of cholesterol and neutral lipids in parenchymal cells. This steatotic phenotype was associated with liver injury, as revealed by serum AST and ALT activities (Figure 1(d)).

### 3.2. Cholesterol Overload Sensitizes to BDL-Induced Liver Damage and Aggravates Obstructive Jaundice

**Figure 2:**
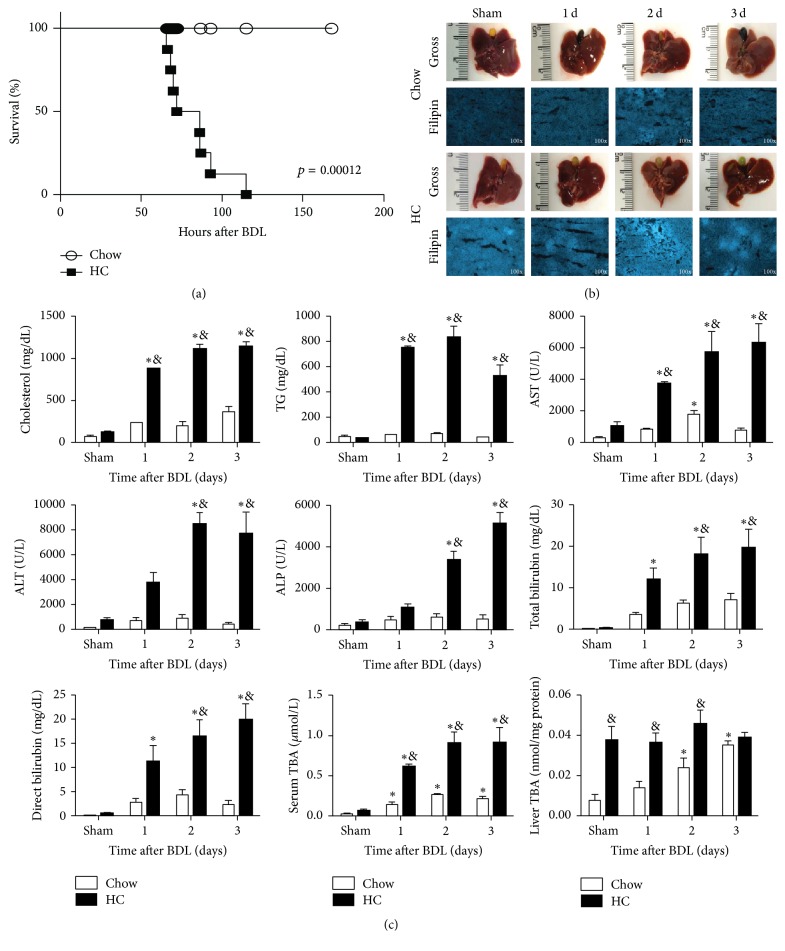
Cholesterol overload sensitizes to BDL-induced liver damage and aggravates obstructive jaundice. (a) Survival plot after BDL; at least nine animals were considered for the survival experiment. (b) Liver gross inspection and free cholesterol determination by filipin. Images are representative of at least three independent experiments. Original magnification for filliping staining: 100x. (c) Biochemical serum cholesterol and triglycerides (TG) determination. Liver function test: aspartate aminotransferase (AST); alanine aminotransferase (ALT); alkaline phosphatase (ALP); total bilirubin; direct bilirubin; serum total bile acids (TBA); and liver tissue TBA. Each column represents mean ± SEM of three independent experiments. Differences were considered significant at ^*∗*^ *p* ≤ 0.01 versus Sham Chow and ^&^ *p* < 0.01 versus Chow fed mice at the same time.

We next examined the impact of HC feeding in BDL-induced liver injury and survival. Mice fed with the HC diet and subjected to BDL surgery died between 60 and 115 h after procedure, compared to mice fed with control Chow diet (Figure 2(a)); none of the HC fed mice survived beyond 5 days after BDL. Although BDL is a model for acute obstructive cholestasis, control mice survived up to 6 weeks after surgery, in agreement with previous results [19, 20].

As a high percentage of HC fed mice died within 3 days after surgery, we decided to characterize liver function at 1, 2, and 3 days after BDL to assess the mechanisms contributing to the early death of HC fed mice.

Liver gross inspections revealed no significant differences between groups; mice presented bile duct stasis and greenish gallbladder in HC group, in comparison with Chow animals, which exhibited a darker gallbladder (Figure 2(b)). Cholesterol content, assayed by filipin staining, increased in HC animals. At day 3 cholesterol content was less diffused in the tissue, displaying spots with high fluorescence, while Chow fed animals liver cholesterol content was uniform and unchanged following BDL.

Serum cholesterol and TG content were also elevated in HC fed mice and remained increased after BDL. Liver injury and function tests exhibited a significant increase in HC fed mice since day one after BDL in AST and ALT and at two days in ALP. In all cases, values raised until day three in HC fed mice. In contrast, Chow fed animals showed a slight increase at day two in AST, and no significant changes were observed up to the third day.

Figure 2(c) depicts the main biochemical tests to address liver function and damage. Total and free bilirubins were also increased in HC fed animals since the first day of surgery, and a similar effect was observed in serum total bile acids (TBA). Interestingly, liver TBA were significantly augmented in Sham animals due to HC feeding with no changes in the course of the study, while Chow fed animals exhibited significantly increased liver TBA at day two, peaking at the third day.

No significant changes were found in mice fed with a diet supplemented with 0.5% sodium cholate alone, as control diet, and subjected to BDL comparing with Chow diet fed animals.

In order to figure out the mechanism that could explain the premature death in HC fed animals, we focused on studying the phenotype and molecular changes at day three.

**Figure 3:**
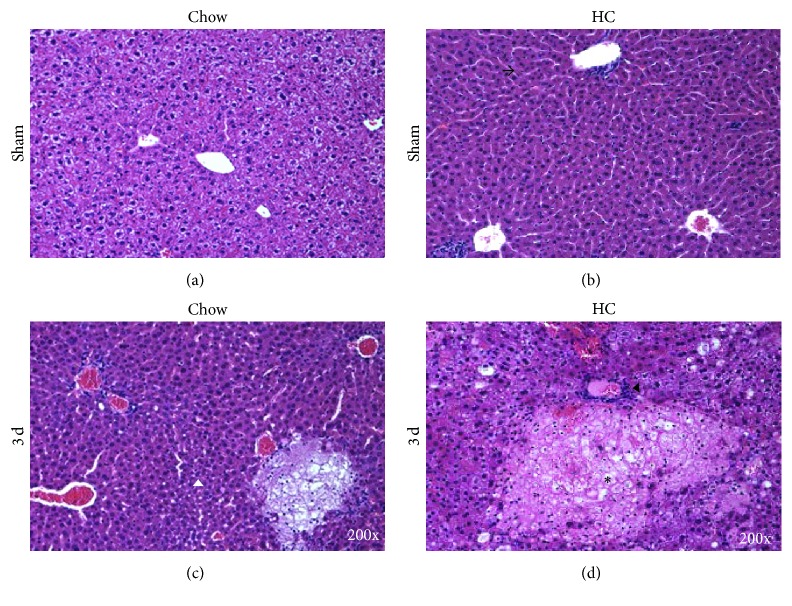
Cholesterol overload exacerbates liver tissue damage. Histology analysis by H&E staining. Liver tissue was obtained from animals fed with standard Chow control diet (Chow) or high cholesterol (HC) diet for 2 days and subjected to BDL. Bridging hepatic necrosis areas (asterisks), polymorphonuclear leukocytes and lymphocytes infiltration (black arrow head), inflammatory cells infiltration (arrow), and cell proliferation (white arrow head). Representative images from at least four different animals. Original magnification: 200x.

Histological analysis of livers from HC fed mice (Figure 3) revealed lymphocytes (black arrow head) infiltration and prominent bridging hepatic necrosis seen in HC fed animals comparing with Chow fed animals, which presented this characteristic to a lesser extent. Chow fed animals presented evidence of cell proliferation (white arrow head), suggesting an ongoing repair process.

### 3.3. HC Feeding Decreases Cell Proliferation following BDL

**Figure 4:**
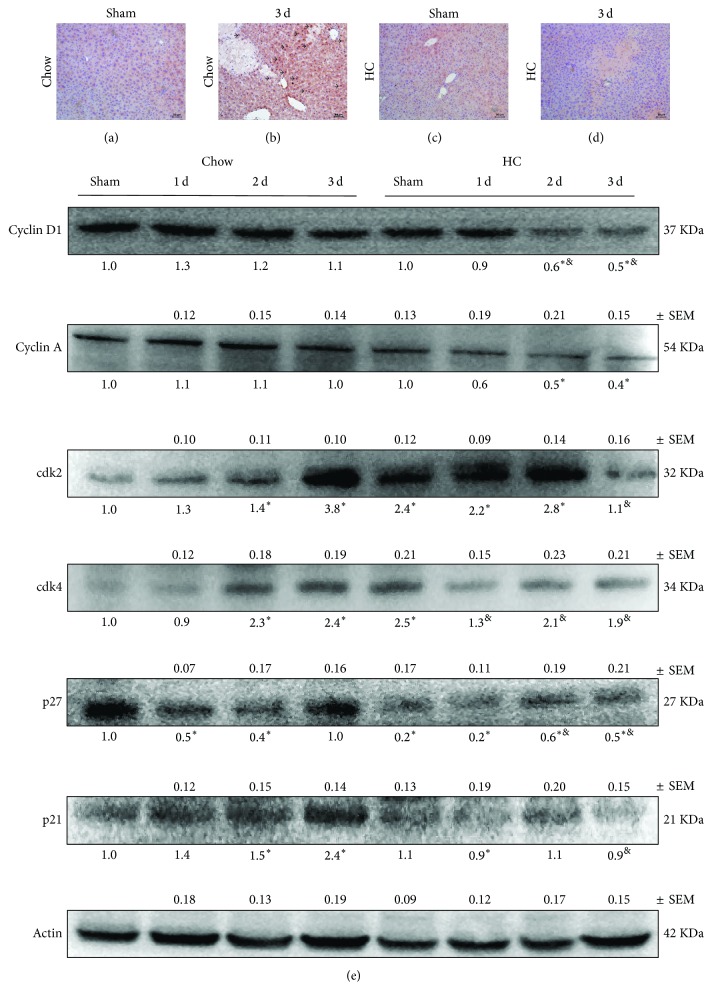
Cholesterol excess impairs cell proliferation. Tissue was obtained from Sham and three days after BDL animals, under (a-b) standard Chow control diet (Chow) or (c-d) high cholesterol (HC) diet, and Ki67 immunohistochemistry was assayed; arrows show positive cells (magnification at 100x). (e) Western blot and densitometric analysis of main cell cycle proteins. Actin was used as loading control; ^*∗*^ *p* < 0.05 versus Sham Chow fed mice, ^&^ *p* < 0.05 versus Sham HC fed mice. Images are representative from at least four independent mice.

In order to address whether cholesterol loading interfered with cell proliferation during BDL, as suggested by H&E staining, we performed Ki67 staining in samples from both groups of mice at the third day after BDL.  Figure 4(b) shows more positive Ki67 cells (black arrows) in liver samples from Chow fed mice than liver from HC fed mice (Figure 4(d)). Results were in agreement with the expression of the main cell cycle proteins cyclins D1 and A and cdk2 and cdk4, showing a sustained expression in a time-dependent manner in Chow fed animals, particularly in cdk2 and cdk4, which decreased in HC mice, particularly at day three. Cell cycle inhibitory proteins, such as p27 and p21, presented different comportment; p27 increased in time-dependent manner, but p21 decreased (Figure 4(e)).

### 3.4. BDL in HC Fed Mice Induces Apoptosis and Oxidative Stress

**Figure 5:**
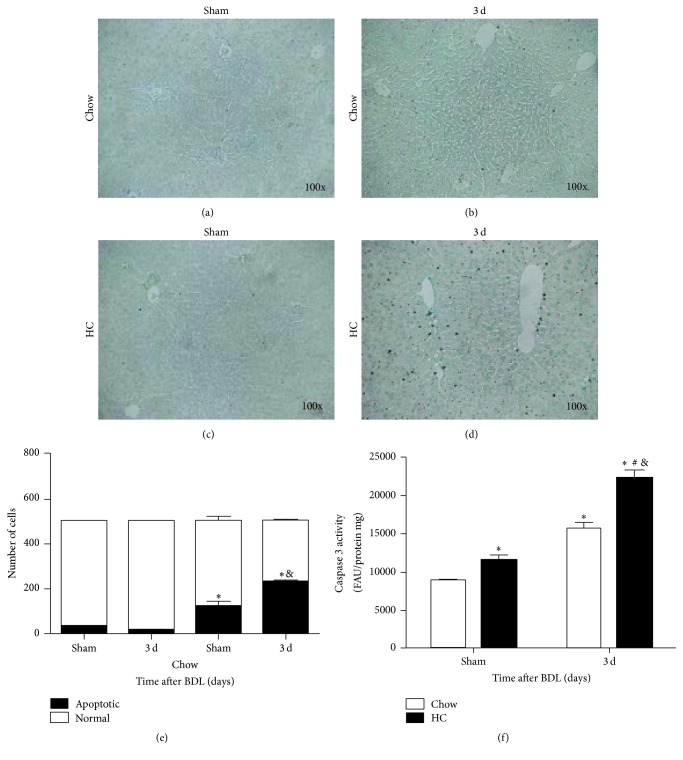
BDL in HC fed mice increases apoptosis. Tissue was obtained from Sham and three days after BDL animals, under (a-b) standard Chow control diet (Chow) or (c-d) high cholesterol (HC) diet, and apoptosis was assayed by (e) TUNEL immunohistochemistry; (e) quantification of positive TUNEL cells or by (f) caspase 3 activity. Images are representative from at least four independent mice. Original magnification: 100x. ^*∗*^ *p* < 0.01 versus Chow fed mice, ^#^ *p* < 0.01 versus 3 days Chow fed mice, and ^&^ *p* < 0.01 versus HC fed mice.

It is well known that bile salts' accumulation in the liver induces apoptosis in hepatocytes [21, 22].  Figure 5 shows that HC fed mice subjected to BDL surgery presented more apoptotic cells, assayed by TUNEL (Figure 5(d)), than Chow fed animals (Figure 5(b)). The quantification of apoptotic cells revealed a significant increment in positive cells in HC fed animals (Figure 5(e)). These data were corroborated by measurement of caspase 3 activity (Figure 5(f)).

**Figure 6:**
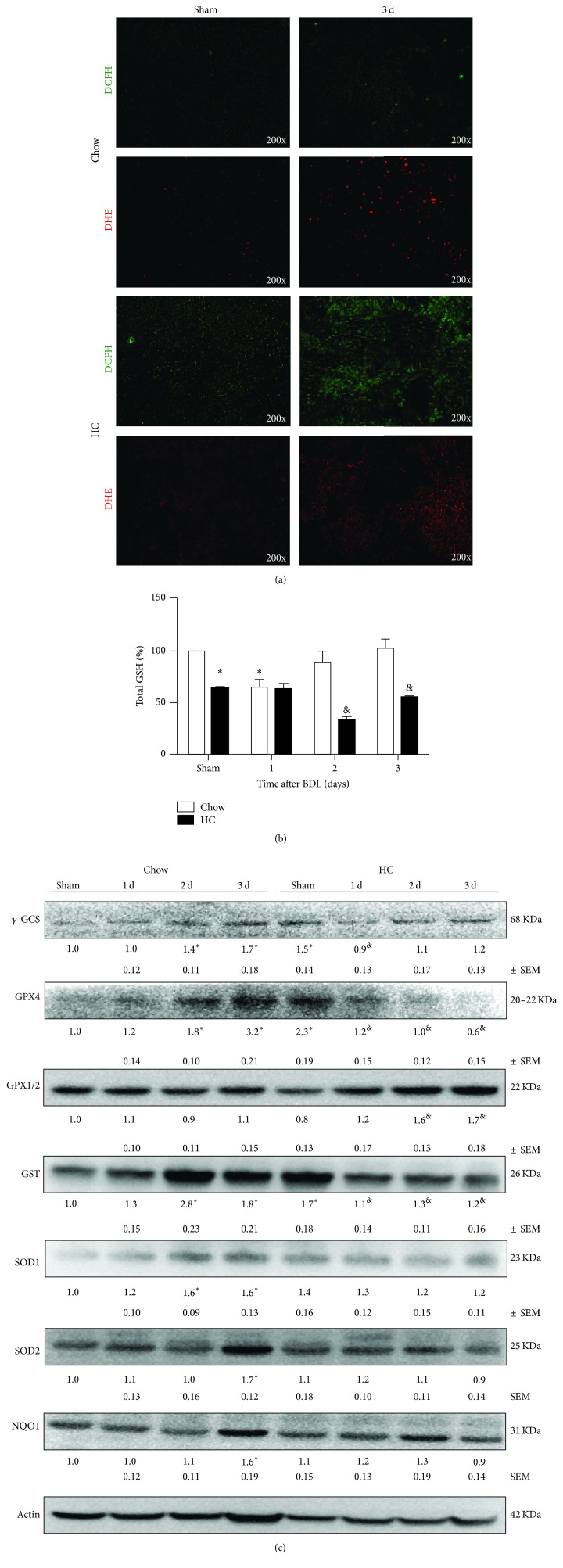
HC diet increases* oxidative stress in mice with BDL*. (a) ROS content determined in fresh liver sections. Peroxides content was assayed by DCFH (5 *μ*M) for 30 min, and superoxide content was determined by DHE (50 *μ*M) for 30 min. (b) GSH content determined by HPLC. (c) Western blot and densitometric analysis of main oxidative stress-related proteins. Actin was used as loading control. Each column represents mean ± SEM of three independent experiments. Differences were considered significant at ^*∗*^ *p* ≤ 0.01 versus Sham Chow fed animals; ^&^ *p* ≤ 0.01 versus Sham Chow fed animals at the same time. Images are representative of at least three independent experiments. Original magnification: 200x.

Oxidative stress is one of the major inducers of hepatic apoptosis [23]. Therefore, we measured the* in situ* content of ROS by DCFH and DHE, as previously reported [18]. As seen, DCFH and DHE staining increased in livers sections from HC fed animals at day 3 after BDL, compared to Chow fed animals (Figure 6(a)); these results were in agreement with a decrease in total GSH levels (Figure 6(b)). GSH levels decreased as a result of HC feeding and continued to decline after BDL.

Importantly, Chow fed animals exhibited decreased GSH content at day one of treatment, which subsequently augmented at day two reaching basal GSH levels at the third day. The analysis of the main antioxidant enzymes (Figure 6(c)) revealed an induction of *γ*-GCS and GPX4 in Chow fed animals at days two and three, an effect that was not observed in HC animals; even more, GPX4 decreased in HC fed animals in time-dependent manner. GPX1/2 were slightly induced in HC fed mice. GST exhibited a time-dependent increment in Chow fed animals, with opposite effect in HC fed mice. Other antioxidant enzymes such as SOD1, SOD2, and NQO1 showed an increment in Chow fed mice but not so in HC fed mice; even more, SOD2 showed a decrease in the time of the study in HC fed animals.

## 4. Discussion 

Bile duct obstruction-mediated liver injury is a key feature of cholestasis, which underlies several chronic liver diseases, such as gallstone impaction, biliary atresia, and tumor compression [24]. The accumulation of toxic bile acids during bile duct obstruction contributes to the hepatocellular injury. The mechanism of damage is complex and involves oxidative stress generation and inflammation [14, 24, 25].

It is well known that bile acids arise from enzymatic oxidation of cholesterol and that hypercholesterolemia leading to hepatic cholesterol accumulation is one of the key hallmarks in NAFLD and NASH [8, 10]. Consumption of cholesterol-enriched diets could aggravate NAFLD, particularly in susceptible individuals related to congenital risks or deficient dietary habits [26, 27]. For instance, in Hispanics, the consumption of high cholesterol diets is very common, which may account for the high prevalence of NAFLD in this population [28].

In the present work we used a nutritional model of hepatic cholesterol overload to examine the impact of cholesterol in obstructive cholestasis-mediated liver injury. Mice fed a HC-enriched diet for two days showed a steatotic phenotype with particular lipid overload in hepatocytes, comprising primarily increased cholesterol accumulation (2-3-fold), and exhibited liver damage, as revealed by serum AST and ALT values and histology examination. Although HC diet was supplemented with 2% cholesterol, HC fed mice exhibited a mild increase in TG content in hepatocytes. Whether TG accumulation reflects increased FFA esterification into TG or impairment in mitochondrial *β*-oxidation due to cholesterol trafficking to mitochondria, as we reported previously [4], remains to be further established.

In line with the emerging role of cholesterol in NAFLD progression to NASH in mice and humans and the role of cholesterol in liver fibrogenesis, we show the sensitization of mice fed HC diet to BCL-mediated liver injury and premature death. Moreover, cholesterol overload exacerbates BDL-induced deterioration of liver function, including increased total and free bilirubin and TBA in serum since day one after BDL, but not in Sham animals, indicating that this response is a consequence of bile duct obstruction. Interestingly, hepatic TBA content increased as a result of HC feeding, with no significant further increment following BDL as opposed to chow fed mice, which exhibit a time-dependent increase in liver TBA, reaching similar values observed in HC fed animals at day three. The toxic effect of bile acids is well documented [21, 29, 30] and accumulation in the liver is one of the main factors associated with cholestasis.

Besides accumulation of toxic bile acids, our data suggest that cholesterol overload impairs cell proliferation and tissue repair as revealed by Ki67 immunostaining. This response is accompanied by decreased p21 expression levels in cholesterol loaded livers and a compensatory increased expression of cdk2. We observed that HC fed mice exhibit an increased content of total bile acids, which are associated with cell death. These findings suggest that cholesterol antagonizes the reported protective effect mediated by FXR activation. In support of this link, Cheng and coworkers recently reported that FXR transgenic mice exhibited increased sensitivity to a high cholesterol diet-induced hepatotoxicity [31], indicating that FXR is unable to protect from cholesterol-mediated liver injury.

On the other hand, it is well known that oxidative stress mediates the deleterious effects of toxic bile acids and cholesterol [9, 21, 25, 32]. Our findings are in agreement with these observations, as HC fed mice exhibit increased DCFH and DHE staining following BDL compared to Chow fed mice. In line with previous findings linking cholesterol accumulation in the liver with mitochondria-mediated ROS generation and impairment in antioxidant defense, we observed the downregulation of GPX4 and SOD2, two of the main mitochondrial protective enzymes [17, 33] in HC fed mice after BDL. Moreover, GST and *γ*-GCS display a similar behavior, strongly supporting the fact that the disruption in GSH homeostasis is an important contributor to cholesterol-mediated hepatocellular damage.

It is clear that cholesterol overload in the liver aggravates the organ injury decompensating GSH system, which could be related to a rapid cell death, particularly by apoptosis, potentiated by the overproduction of bile salts that eventually conducts to organ failure and animal death. Animals subjected to sodium cholate alone diet do not exhibit any significant change during the five days of the study.

It is clear that liver with cholesterol overload is sensitized to damage aggravating the diseases; this could be particularly considered in patients with genetic susceptibility to cholesterol overload, such as early infantile forms of Niemann-Pick C disease, where canonical liver insults must be under surveillance.

Overall, our findings may be of clinical relevance suggesting that patients with cholesterol liver overload can be more at risk of developing clinical complications associated with cholestasis. In this context the use of statins may be of potential benefit by targeting the mevalonate pathway and perhaps other noncholesterol related mechanisms involved in cholestasis-mediated liver injury.

## Supplementary Material

Supplementary figure 1. A) Experimental design for the animal studies. B) Liver gross inspection from animals fed with control diet supplemented with sodium cholate 0.5% (Cholate), or with the high cholesterol diet (HC) for 2 and 30 days. C) Liver/Body weight ratio, AST and ALT serum activities of animals under cholate (0.5%) alone diet or HC for 2 and 30 days. Each column represents mean ± SEM of three independent experiments. Differences were considered significant at ∗ p ≤ 0.05 vs Chow animals.
Supplementary figure 2. Antibodies used in the study.
Supplementary figure 3. A high cholesterol diet induces hepatocyte free cholesterol and neutral lipids overload. Hepatocytes were isolated from animals fed with Standard control diet (Chow) or High cholesterol diet (HC). A-B) Free cholesterol determination by filipin, C-D) Neutral lipid determined by Oil Red O staining (ORO), Biochemistry determination of E) total cholesterol; and F) triglycerides (TG) content. Differences were considered significant at ∗ p ≤ 0.01 vs Chow. Images are representative of at least three independent experiments. Original magnification 200X.
