# Supplementary material for: Liver Cholesterol Overload Aggravates Obstructive Cholestasis by Inducing Oxidative Stress and Premature Death in Mice
Source: Oxid Med Cell Longev. 2016 Aug 21;2016:9895176. doi: 10.1155/2016/9895176 (PMC5011220; doi:10.1155/2016/9895176)
Supplement: Supplementary file 1 — Supplementary figure 1. A) Experimental design for the animal studies. B) Liver gross inspection from animals fed with control diet supplemented with sodium cholate 0.5% (Cholate), or with the high cholesterol diet (HC) for 2 and 30 days. C) Liver/Body weight ratio, AST and ALT serum activities of animals under cholate (0.5%) alone diet or HC for 2 and 30 days. Each column represents mean ± SEM of three independent experiments. Differences were considered significant at ∗ p ≤ 0.05 vs Chow animals. Supplementary figure 2. Antibodies used in the study. Supplementary figure 3. A high cholesterol diet induces hepatocyte free cholesterol and neutral lipids overload. Hepatocytes were isolated from animals fed with Standard control diet (Chow) or High cholesterol diet (HC). A-B) Free cholesterol determination by filipin, C-D) Neutral lipid determined by Oil Red O staining (ORO), Biochemistry determination of E) total cholesterol; and F) triglycerides (TG) content. Differences were considered significant at ∗ p ≤ 0.01 vs Chow. Images are representative of at least three independent experiments. Original magnification 200X. [file 9895176.f1.docx]

**Liver cholesterol overload aggravates obstructive cholestasis by inducing oxidative stress and premature death in mice**

**SUPPLEMENTARY MATERIAL**

**Materials and Methods**

*Hepatocyte isolation*

Hepatocytes were isolated from Chow and HC diet fed mice by the two-step collagenase perfusion, as we previously described (Clavijo-Cornejo, 2013). The viability was >90% as assessed by trypan blue exclusion. Hepatocytes were seeded at 2.13 × 10^5^ cells per cm^2^ in Lab-Tek chambered slides (Nalge, Nunc) in the Ham's F-12/Dulbecco's modified Eagle's basal hepatocyte growth medium supplemented with 10% fetal bovine serum. After a 4 h attachment, the medium was replaced to a serum-free basal hepatocyte growth medium. The following day, cells were treated with 50 ng/ml HGF.

**Supplementary figures**

**A)**

**
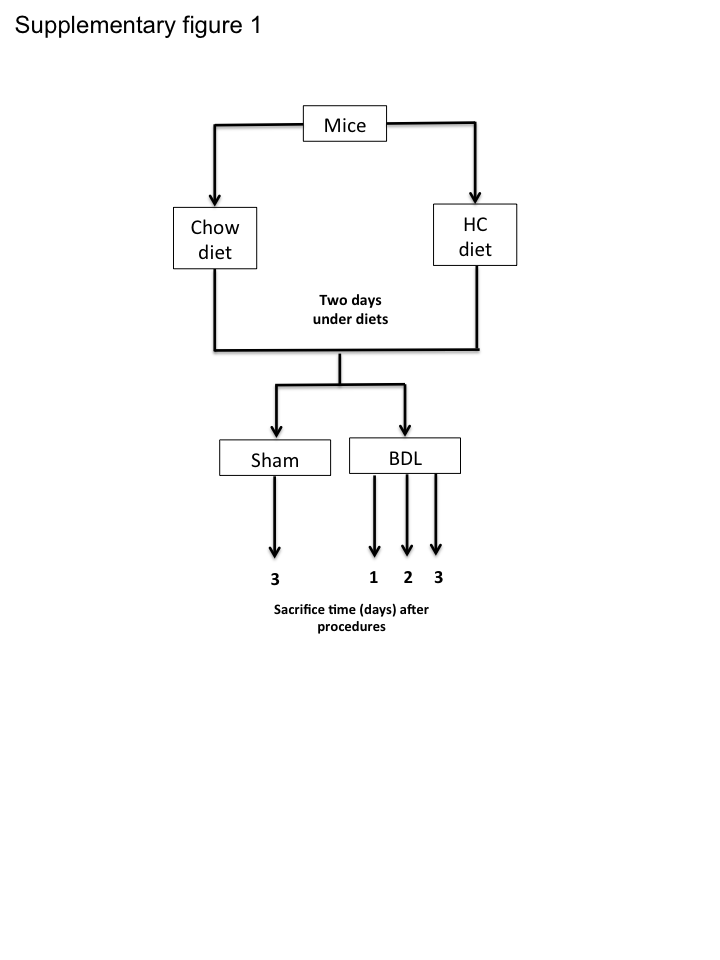
**

**
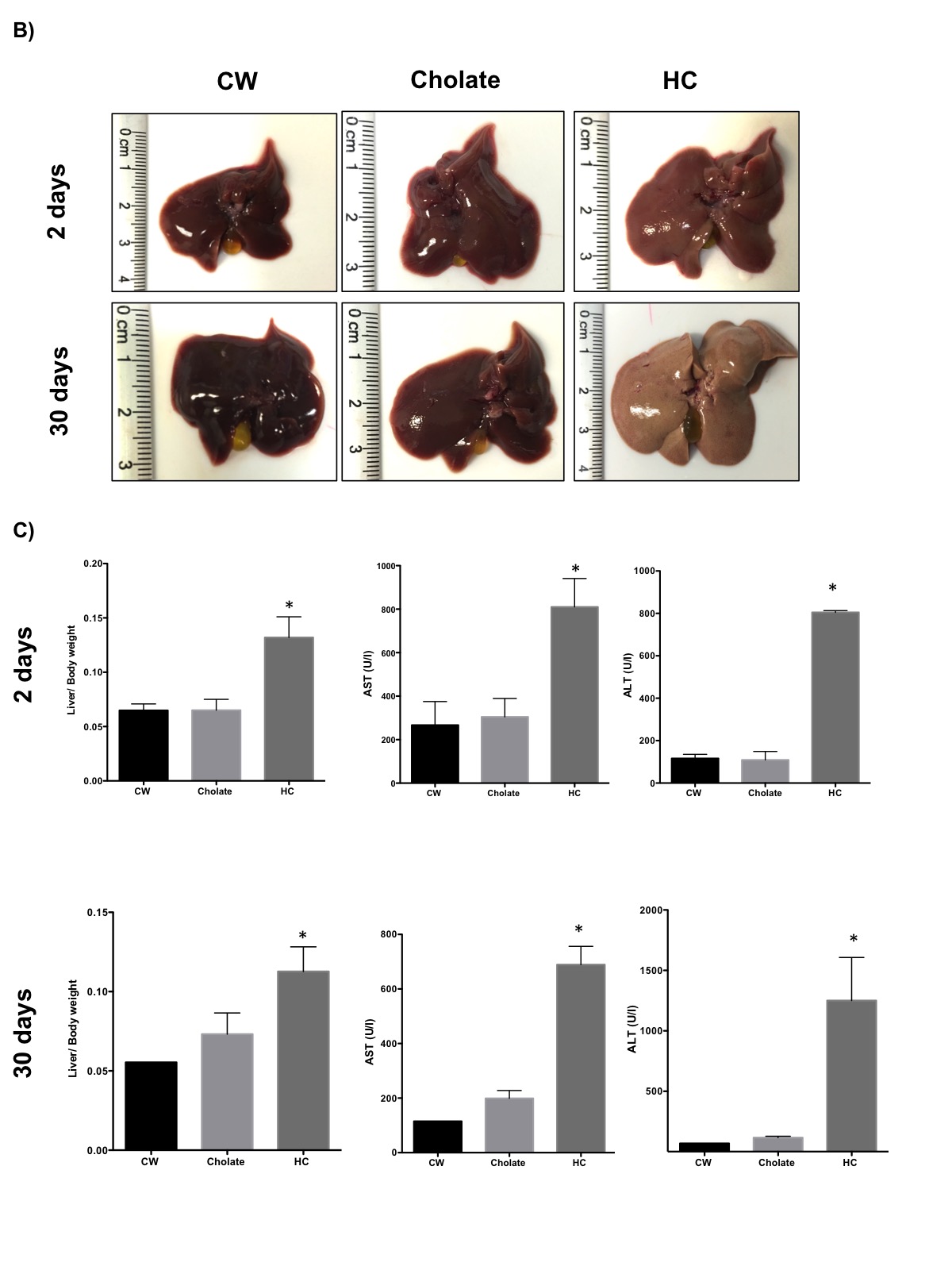
**

**Supplementary figure 1.** A) Experimental design for the animal studies. B) Liver gross inspection from animals fed with control diet supplemented with sodium cholate 0.5% (Cholate), or with the high cholesterol diet (HC) for 2 and 30 days. C) Liver/Body weight ratio, AST and ALT serum activities of animals under cholate (0.5%) alone diet or HC for 2 and 30 days. Each column represents mean ± SEM of three independent experiments. Differences were considered significant at * p ≤ 0.05 vs Chow animals.

**
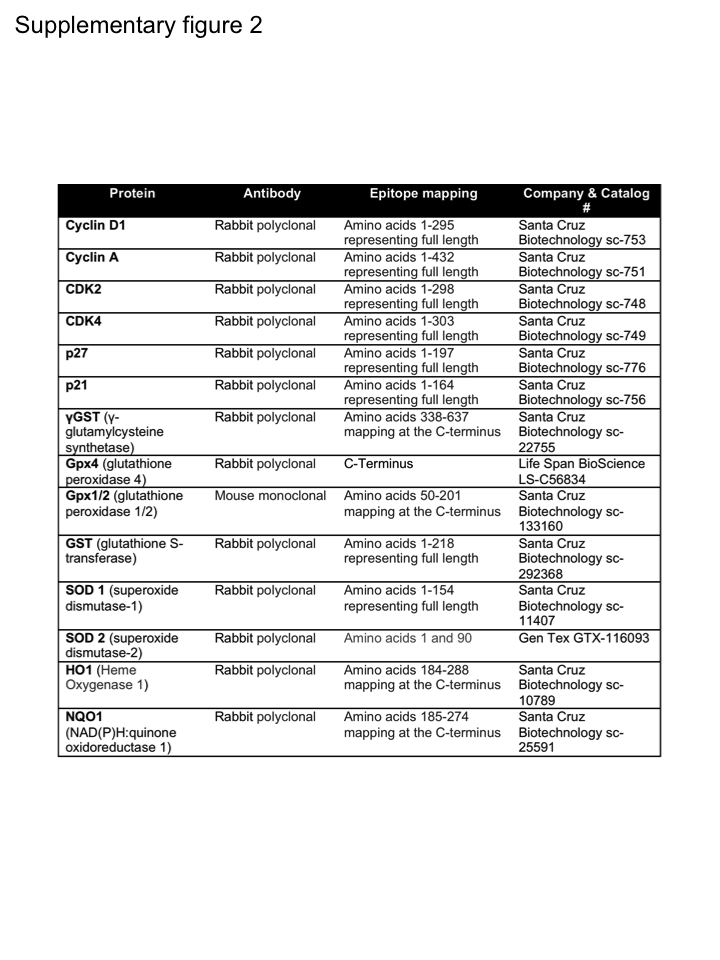
**

**Supplementary figure 2. Antibodies used in the study.**

**
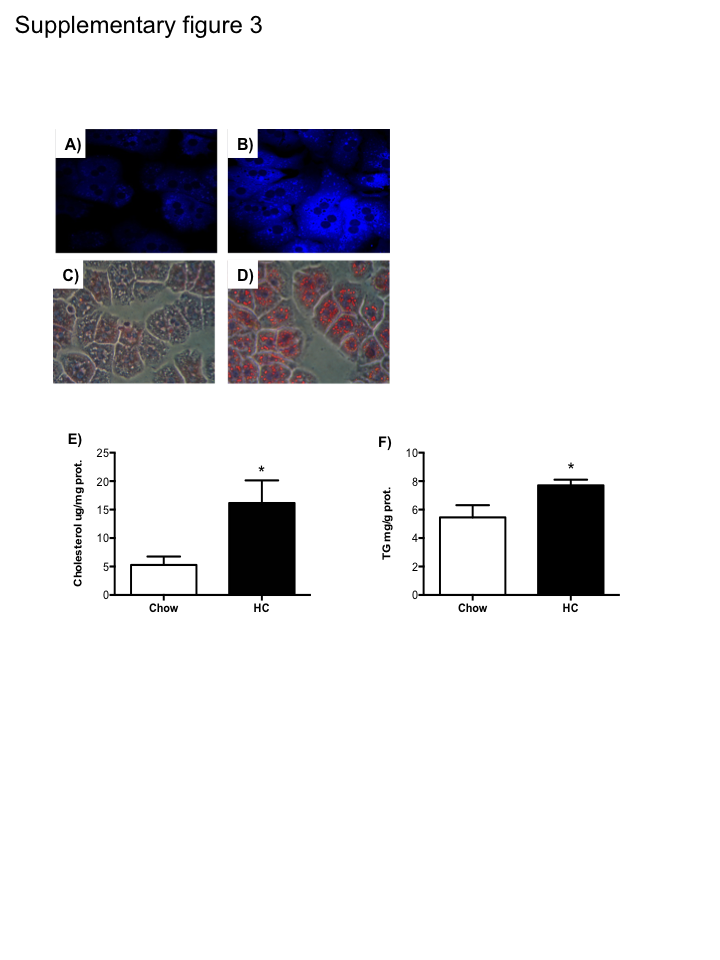
**

**Supplementary figure 3. A high cholesterol diet induces hepatocyte free cholesterol and neutral lipids overload.** Hepatocytes were isolated from animals fed with Standard control diet (Chow) or High cholesterol diet (HC). A-B) Free cholesterol determination by filipin, C-D) Neutral lipid determined by Oil Red O staining (ORO), Biochemistry determination of E) total cholesterol; and F) triglycerides (TG) content. Differences were considered significant at * p ≤ 0.01 vs Chow. Images are representative of at least three independent experiments. Original magnification 200X.
